# The Effect of Exclusive Olive Oil Consumption on Successful Aging: A Combined Analysis of the ATTICA and MEDIS Epidemiological Studies

**DOI:** 10.3390/foods8010025

**Published:** 2019-01-12

**Authors:** Alexandra Foscolou, Elena Critselis, Stefanos Tyrovolas, Christina Chrysohoou, Labros S. Sidossis, Nenad Naumovski, Antonia-Leda Matalas, Loukianos Rallidis, Evangelos Polychronopoulos, Jose Luis Ayuso-Mateos, Jose Maria Haro, Demosthenes Panagiotakos

**Affiliations:** 1 Department of Nutrition and Dietetics, School of Health Science and Education, Harokopio University, 17676 Athens, Greece; alexandra.foscolou@gmail.com (A.F.); ecritsel@hua.gr (E.C.); s.tyrovolas@pssjd.org (S.T.); lss133@rci.rutgers.edu (L.S.S.); amatala@hua.gr (A.-L.M.); evpol@hua.gr (E.P.); 2 Parc Sanitari Sant Joan de Déu, Fundació Sant Joan de Déu, CIBERSAM, Universitat de Barcelona, 08830 Barcelona, Spain; jmharo@pssjd.org; 3 Instituto de Salud Carlos III, Centro de Investigación Biomédica en Red de Salud Mental, CIBERSAM, Monforte de Lemos 3-5. Pabellón 11, 28029 Madrid CIBER of Mental Health, 28029 Madrid, Spain; joseluis.ayuso@uam.es; 4 First Cardiology Clinic, School of Medicine, University of Athens, 11527 Athens, Greece; chrysohoou@usa.net; 5 Department of Kinesiology and Health, School of Arts and Sciences, Rutgers University, New Brunswick, NJ 08901, USA; 6 Faculty of Health, University of Canberra, ACT 2617 Canberra, Australia; Nenad.Naumovski@canberra.edu.au; 7 Second Cardiology Clinic, School of Medicine, University of Athens, 12462 Athens, Greece; lrallidis@gmail.com; 8 Hospital Universitario de La Princesa, Instituto de Investigación Sanitaria Princesa (IP), 28006 Madrid, Spain; 9 Department of Psychiatry, Universidad Autonoma de Madrid, 28049 Madrid, Spain

**Keywords:** olive oil, successful aging, dietary fats

## Abstract

The consumption of dietary fats, which occur naturally in various foods, poses important impacts on health. The aim of this study was to elucidate the association of exclusive use of olive oil for culinary purposes with successful aging in adults aged >50 years old and residing in Greece. Use of olive oil in food preparation and bio-clinical characteristics of the Greek participants enrolled in the ATTICA (*n* = 1128 adults from Athens metropolitan area) and the MEDiterranean Islands Study (MEDIS) (*n* = 2221 adults from various Greek islands and Mani) studies, were investigated in relation to successful aging (SA). Participants were divided into the following three categories: (a) no olive oil consumption; (b) combined consumption of olive oil and other dietary fats; and (c) exclusive olive oil consumption. The SA was measured using the previously validated successful aging index (SAI). After adjusting for age, sex, and smoking habits, combined consumption of olive oil and other fats (vs. no olive oil use) was not significantly associated with SAI levels (*p* = 0.114). However, exclusive olive oil intake (vs. no use of olive oil) was significantly associated with SAI (*p* = 0.001), particularly among those aged older than 70 years. Therefore, the exclusive consumption of olive oil, as opposed to either combined or no olive oil consumption, beneficially impacts successful aging, particularly among individuals over 70 years of age. Primary public health prevention strategies should seek to encourage the enhanced adoption of such dietary practices in order to promote healthy aging and longevity.

## 1. Introduction

Dietary fats occur naturally in various foods and their excessive consumption negatively impacts human health; dietary fat consumption can confer benefits, albeit also adverse effects concerning the onset and progression of several nutrition-related chronic diseases [1]. The use of fats of either plant or animal origin is embedded in practically every food culture as fats provide energy and lipid soluble vitamins, facilitate food preservation, and greatly contribute to food’s sensorial characteristics. Amongst dietary fats, the consumption of plant oils is considered to render the greatest health benefits as they contain a higher proportion of unsaturated fatty acids compared to animal fats [2]. Thus, plant oils are a vital and beneficial source of energy. The most commonly consumed types of plant oils include palm, soybean, canola, sunflower-seed, and, most prominently, olive oil [3]. 

Olive oil in particular is an essential component of the Mediterranean diet and its frequent consumption is associated with a multitude of health benefits [4], including the deterrence of cardiovascular diseases [5], diabetes mellitus [6], metabolic syndrome [7], and several types of cancers [8]. Therefore, the differential impact of olive oil consumption, as compared to the ingestion of other plant oils, has been gaining increasing interest. A relatively recent meta-analysis of 32 cohorts (*n* = 841,211) demonstrated that the higher consumption of monounsaturated fatty acids (highest vs. lowest third) is associated with lower cardiovascular mortality (relative risk (R.R.): 0.88, 95% CI: 0.80–0.96), stroke (R.R.: 0.83, 95% CI: 0.71–0.97), and all-cause mortality (R.R.: 0.89, 95% CI: 0.83–0.96). It is noteworthy, though, that subgroup analyses revealed that these effects were attributed primarily to olive oil consumption, and not to the combined consumption of monounsaturated fatty acids arising from both plant and animal origins [9]. In addition, preliminary findings reveal that the frequent consumption of olive oil may also beneficially impact the aging process [10,11].

Aging is a multifactorial process involving different alterations regarded as the "hallmarks of aging". These hallmarks include genomic instability, telomere attrition, epigenetic alterations, loss of proteostasis, deregulated nutrient sensing, mitochondrial dysfunction, cellular senescence, stem cell exhaustion, and altered intracellular communication [12]. These hallmarks are influenced by olive oil intake, among other factors. Specifically, several of such effects may be accounted for by oleic acid (a main constituent of olive oil) and the bioactivity of its minor compounds (such as polyphenols), which as a result of their ability to modulate gene expression, may affect cellular aging through both direct and indirect mechanisms. In fact, one of the major phenol classes present in olive oil, secoiridoids, exhibits the capacity to modulate several pathways entailed in aging. These beneficial effects have been observed in both in vitro and in vivo animal models [13] as well as in human studies, revealing the potential beneficial effects of olive oil intake on the aging process [12].

Although olive oil and its compounds have beneficial effects on aging at the cellular level, there is still a lack of evidence regarding the role of olive oil in the concept of successful aging at a human level. Thus, the aim of this study was to elucidate the association of exclusive consumption of olive oil with successful aging, in people over the age of 50—a period of subtle changes in the human body—living in Greece.

## 2. Materials and Methods

The data from two cross-sectional, population-based, large-scale epidemiologic studies (namely the ATTICA [14] and the MEDIS (MEDiterranean Islands Study) [15] studies), conducted in Greece, were combined for the purposes of the present investigation. 

### 2.1. Study Sample

Data from both the ATTICA and the MEDIS studies included the information collected from adults aged over 50 years living in urban and insular Greek areas. The ATTICA study was a population-based observational study implemented in the greater metropolitan Athens area in Greece during 2001–2002. The main aims of this study were to investigate the prevalence of cardiovascular disease risk factors, potential predictor factors of cardiovascular disease (CVD), and the 10-year (2002–2012) incidence of the disease [14]. At baseline, all participants were free of CVD and cancer, as assessed through a detailed clinical evaluation by the study’s physicians. From the original sample aged >18 years (*n =* 2583), a sub-group of *n* = 1128 individuals aged >50 years old were analyzed for the purposes of the present work. Additionally, approximately 3000 older people from Mani (Greek peninsula region) and 26 Mediterranean islands of 5 countries were enrolled during 2005–2017 (MEDIS study). Individuals who resided in assisted-living centers, had a clinical history of CVD or cancer, or had left the island for a considerable period of time during their life (i.e., >5 years) were excluded. From the *n* = 3138 participants over 50 years old living in the insular Mediterranean region, of the MEDIS study, a sub-group of *n* = 2221 individuals from 20 Greek islands only were analyzed in this work. More information about the MEDIS study may be found elsewhere [15]. In both studies, a group of health scientists (cardiologists, general practitioners, physicians, dietitians, public health nutritionists, and nurses) with field experience collected all required information using standard, validated questionnaires and clinical procedures. 

### 2.2. Bioethics

The ATTICA study was approved by the Bioethics Committee of Athens Medical School and was carried out in accordance with the Declaration of Helsinki (1989) of the World Medical Association. The MEDIS study followed the ethical recommendations of the World Medical Association (52nd WMA General Assembly, Edinburgh, Scotland, October 2000). The Institutional Ethics Board of Harokopio University approved the study design (16/19-12-2006). In both studies, participants were informed about the aims and procedures of the study and provided written informed consent prior to their enrollment.

### 2.3. Measurements

#### 2.3.1. Sociodemographic Data

The sociodemographic characteristics used in this study included age (years), gender (male/female), and smoking status. Current smokers were defined as those who smoked at least one cigarette or any type of tobacco per day at the time of the interview. Former smokers were defined as those who previously smoked but had quit within the previous year. Current and former smokers were combined as ever smokers. The remaining participants were defined as nonsmokers.

#### 2.3.2. Physical Activity Levels

Physical activity was evaluated in metabolic equivalent (MET)-minutes per week, using the shortened, translated, and validated in Greek version of the self-reported International Physical Activity Questionnaire (IPAQ) [16]. Those who reported at least 3 MET-minutes per week were classified as physically active. All others were defined as physically inactive.

#### 2.3.3. Anthropometric and Clinical Characteristics

In both studies, weight and height were measured using standard procedures to attain the volunteer’s body mass index (BMI) (kg/m^2^). Overweight was defined as BMI between 25.0 and 29.9 kg/m^2^, while obesity was defined as BMI >29.9 kg/m^2^. Waist circumference was measured in the middle between the lowest rib and the iliac crest, using an inelastic measuring tape, to the nearest 0.5 cm. Waist-to-hip ratio was also calculated. Type 2 diabetes mellitus was determined by measuring fasting plasma glucose in accordance with the American Diabetes Association diagnostic criteria (fasting blood glucose >126 mg/dL or use of antidiabetic medication). Participants who had blood pressure levels >140/90 mmHg or who used antihypertensive medications were classified as hypertensive. Fasting blood lipid levels (including high-density lipoprotein-cholesterol (HDL), low-density lipoprotein-cholesterol (LDL), and triglycerides (TG)) were also recorded. Hypercholesterolemia was defined as total serum cholesterol levels >200 mg/dL or the use of lipid-lowering agents, according to the National Cholesterol Education Program Adult Treatment Panel III guidelines [17]. The coefficient of variation for the blood measurements was less than 5%. A cumulative variable (range 0–4) indicating the overall burden of known cardiometabolic risk factors (i.e., obesity and history of hypertension, type 2 diabetes, and hypercholesterolemia) was developed; participants having none of the aforementioned risk factors received a score of 0, having one factor a score of 1, etc.

#### 2.3.4. Dietary Habits Assessment

Among ATTICA study participants, the evaluation of dietary habits was based on a semi-quantitative food-frequency questionnaire (FFQ), originally developed for the European Prospective Investigation into Cancer and Nutrition (EPIC) study [18]. The Greek version of the EPIC questionnaire was provided by the Unit of Nutrition of Athens Medical School, after being translated according to standard literature guidelines [19]. All participants were asked to report the average intake (per week or day) of several food items that they had consumed (during the last 12 months). Similar to the ATTICA study, dietary habits in the MEDIS study were assessed through a semi-quantitative, validated, and reproducible FFQ [20]. 

The participants were divided into the following three categories based on the type of dietary fats (raw or cooked) consumed: (a) “No culinary use of olive oil”, defined as consumption of other types of dietary fats, but not olive oil; (b) “Non-exclusive culinary use of olive oil”, defined as the combined consumption of all types of dietary fats; and (c) “Exclusive culinary use of olive oil”, defined as sole consumption of olive oil without examining the specific type of olive oil, e.g., extra-virgin, virgin, or refined olive oil, in any of the three aforementioned groups.

#### 2.3.5. Successful Aging Index

Successful aging index (SAI), with potential scores ranging from 0 to 10, previously developed and validated, was employed to evaluate aging for both ATTICA and MEDIS study participants [15]. The full index encompasses health-related social, lifestyle, and clinical factors, including education, financial status, physical activity, BMI, depression, participation in social activities with friends and family, number of yearly excursions, total number of clinical CVD risk factors (i.e., history of hypertension, diabetes, hypercholesterolemia, obesity), and level of adherence to the Mediterranean diet [15].

#### 2.3.6. Statistical Analysis

Continuous variables were presented as mean ± standard deviation (SD) and categorical variables as frequencies. Associations between continuous variables and group of participants were evaluated with analyses of variance (ANOVA). To correct for the inflation of Type-I errors in multiple comparisons, Bonferroni’s correction was applied. Associations between categorical variables were tested using the calculation of Pearson’s chi-squared test. The association between age and type of consumed oil was tested with Pearson’s correlation coefficient. Linear regression models were used to evaluate the association between categories of dietary oil consumption (namely no use of olive oil versus (a) combined consumption of olive oil and other dietary fats or (b) exclusive consumption of olive oil) and participants’ characteristics (i.e., age, gender, and smoking habits) and the SAI (dependent outcome). Results were presented as unstandardized beta coefficients ± standard error and *p*-value. The STATA software, version 14 (MP & Associated, Sparta, Greece) was used for all statistical analyses. A two-sided *p* < 0.05 was applied as the criterion of significance.

## 3. Results

In Table 1 basic socio-demographic, lifestyle, and clinical characteristics of the participants based on the type of oil are presented. Exclusive culinary olive oil users were more likely to be older (*p* < 0.001), male (*p* < 0.001), physically active (*p* < 0.001), and to have higher adherence to the Mediterranean diet (*p* < 0.001), and they were less likely to have hypertension (*p* = 0.001) compared to non-culinary users of olive oil and/or non-exclusive users.

Moreover, type of oil consumption was directly correlated with age (*r* = 0.15, *p* < 0.001); therefore, a stratified analysis based on the median value of participants’ ages was applied. Participants were divided into two categories, namely one including those aged 50–70 years as compared to those aged ≥70 years old. Table 2 shows that among those aged 50–70 years old, exclusive culinary olive oil users were more likely to be physically active (*p* < 0.001), to have higher levels of adherence to the Mediterranean diet (*p* < 0.001), and less likely to have hypertension (*p* = 0.001). Accordingly, among those aged ≥70 years, exclusive culinary olive oil users were more likely to be physically active (*p* < 0.001), smokers (*p* = 0.002), to have higher level of adherence to the Mediterranean diet (*p* < 0.001) and higher levels of SAI (*p* < 0.001), and they were less likely to have hypertension (*p* = 0.01).

After adjusting for age, gender, and smoking habits, the “Non-exclusive culinary use of olive oil” category in comparison to the “No culinary use of olive oil” category was not significantly associated with SAI levels (*p* = 0.114); only “Exclusive culinary use of olive oil” vs. “No culinary use of olive oil” was significantly associated with SAI (*p* = 0.001) (Table 3). For individuals older than 70 years, there was a positive relationship between “Exclusive culinary use of olive oil” (vs. “No culinary use of olive oil”) and beneficial SAI levels (b ± SE: 0.38 ± 0.15, *p* = 0.01), meaning that people older than 70 years old who consumed exclusively olive oil had higher SAI levels compared to those without any culinary use of olive oil; no significant association was observed for individuals in the 50–70 years old category (*p* = 0.51). It is noteworthy that “Non-exclusive culinary use of olive oil” (vs. “No culinary use of olive oil”) was not significantly associated with SAI levels either in the overall study sample or among the aforementioned age-specific strata (All *p* > 0.05).

## 4. Discussion

The present pooled analysis of two large-scale population-based studies conducted in Greece assessed the impact of olive oil consumption, as a dietary fat in the preparation and cooking of foods, on successful aging. The main study findings reveal that, following adjustment for potential confounding factors including age, sex, and smoking, participants in the “Exclusive culinary use of olive oil” category, defined as the sole consumption of olive oil for food preparation and cooking, were significantly associated with successful aging. The observed beneficial effects were most prominent among individuals aged older than 70 years old. It is noteworthy that combined use of olive oil with other dietary fats during cooking did not exhibit a notable impact on successful aging. Therefore, the exclusive use of olive oil in the preparation and cooking of foods may enhance healthy aging, particularly among the elderly.

One of the potential pathways for explicating the association between exclusive olive oil intake and successful aging can be described via the free radical theory of aging. According to this theory, free radical formation and accumulation can cause damage to biological tissues, leading to the accelerated aging phenotype. However, olive oil is able to reduce free radical production at the mitochondrial level compared to other oils such as the ones rich in polyunsaturated fatty acids [21]. Finally, monounsaturated fatty acids, such as those of olive oil, are positively correlated with longevity, as well as diminishment of age-related morbidities (e.g., cognitive deficits) [21,22]. Within this context, the findings of this study further support the free radical theory of aging (from the epidemiological perspective), exhibiting that higher olive oil intake is associated with successful aging. In addition, similar findings were reported in the previous study of centenarians in Sicily, where participants were most likely to report, among other health promoting factors, high intake of olive oil [23]. Another potential pathway promoting the present findings could be the fact that due to olive oil’s qualities, its habitual use enhances palatability and facilitates a high intake of vegetables [24]. It is found that fruits and vegetables and their antioxidant compounds seem to have beneficial effects in the healthy aging metric [25].

Moreover, it should be noted that olive oil phenols, such as tyrosol, hydroxytyrosol, and oleocanthal, and other important bioactive compounds could be partially responsible for the positive association between olive oil consumption and successful aging. Bioactive compounds of olive oil, e.g., hydroxytyrosol, oleuropein, and tyrosol, have antioxidant and antimicrobial effects [26]. Nutritional attributes vary depending on the different types and sub-types of olive oil. Olive oil’s antioxidant content exhibits a vital role for most of its biological activities, while oleic acid, squalene, and terpenoids have antitumor effects [27]. Based on the review of Giovannelli, it is suggested that the activity of olive oil phenolic compounds has beneficial effects on the aging process, while animal studies support that olive oil phenols can prevent age-related mental and physical dysfunctions [28]. Actually, extra virgin olive oil, due to biophenolic compounds, has been associated with many beneficial effects on diminishing the risk of several diseases, including cardiovascular disease, several cancers, and age-related illness [29].

In developed countries, the continued prolongation of the average human lifespan, albeit with increased chronic disease morbidity rates, incurs continuously increasing healthcare associated costs and emerging public health challenges [30]. Primary public health prevention strategies, such as the promotion of healthy dietary patterns for successful aging, may ultimately deter the onset of chronic disease and healthcare associated costs. As a result, such strategies encouraging the exclusive use of olive oil as a preparatory (unheated) and cooking oil may help improve the sustainability of populations and public health systems alike. However, since it is not yet fully clarified which particular types of olive oils may render the greatest health impacts, further studies are necessary for elucidating whether olive oils with the highest levels of phenolic compound content (namely extra-virgin olive oil) could offer the most health benefits [31]. 

In addition to the benefits of olive oil as an unheated product, it is also worth mentioning that there is a continuous debate on which type of oil or fat in general is best to cook with. It is well known that the higher the level of unsaturation of the fatty acids, the lower the heat stability of the oil; thus, polyunsaturated are better than saturated fats at room temperature, but when they are heated, their structures change and harmful chemicals can appear [32]. Oils rich in polyunsaturated fatty acids, like corn or sunflower oil, generated very high levels of oxidative products (e.g., aldehydes), whereas dietary fats rich in saturated fatty acids (e.g., butter) or monounsaturated fatty acids (e.g., olive oil) produce fewer aldehydes or other potentially dangerous byproducts [33]. According to the USDA (United States Department of Agriculture) extra virgin olive oil—the most popular culinary type of olive oil—contains 73.330 g of monounsaturated fatty acids, 13.330 g of saturated fatty acids, and 6.670 g of polyunsaturated fatty acids per 100 mL [34], and it may be considered as the best oil for consumption [35]. 

### Strengths and Limitations

To our knowledge, this is one of the first studies examining the type of oil consumed in relation to successful aging of older adults, using two different population-based studies. From the epidemiological perspective, using samples from different populations is useful in order to minimize the effect of several types of bias and to multiply the external validity of the findings. In addition, the reported results could be of significant importance for public health policymakers, since a basic principle of empowerment and health promotion is the awareness of people of the effect of harmful behaviors, such as diet related behaviors. This study also has several limitations. The dietary habits, as well as all other participants’ characteristics, were measured once, thus a measurement error may exist and there is always a bias in self-reported questionnaires. Nutrient intake (e.g., type of consumed fat) was calculated through food composition tables using the information retrieved through the FFQs; thus, inaccurate recall of food intake, or a tendency towards social desirability resulting in individuals over-reporting healthy food intake and underreporting unhealthy food intake cannot be ruled out. Moreover, the type of consumed olive oil was not reported; however, the effect of olive oil—regardless of the specific type—consumption on human health is established [36]. Finally, all participants included in this study are from Mediterranean region so extrapolation to other populations and geographical regions may be limited.

## 5. Conclusions

The present findings support that exclusive culinary use of olive oil, as opposed to either non-exclusive or no culinary use of olive oil, beneficially impacts successful aging, particularly among individuals aged over 70 years of age. Therefore, primary public health prevention strategies should encourage the adoption of dietary practices such as consumption of olive oil in order to promote healthy aging and longevity.

## Figures and Tables

**Table 1:** Socio-demographic, lifestyle, and clinical characteristics of the participants based on All and the category type of dietary fat consumption.

	All Participants (*n* = 3349)	No Culinary Use of Olive Oil (*n* = 510)	Non-Exclusive Culinary Use of Olive Oil (*n* = 1687)	Exclusive Culinary Use of Olive Oil (*n* = 1152)	*p* ^0^	*p* ^1^	*p* ^2^	*p* ^3^
Age (years)	69 ± 10	71 ± 9	67 ± 10	73 ± 9	<0.001	<0.001	<0.001	<0.001
Male (%)	52	50	49	59	<0.001	1.000	<0.001	0.003
Ever smokers (% yes)	43	37	44	43	0.030	0.028	1.000	0.076
Physically active (% active)	41	34	36	53	<0.001	1.000	<0.001	<0.001
BMI (kg/m^2^)	28.1 ± 4.4	28.5 ± 3.9	28.2 ± 4.6	27.8 ± 4.4	0.017	0.585	0.127	0.022
Hypertension (% yes)	57	89	89	79	0.001	1.000	<0.001	<0.001
Diabetes (% yes)	21	25	21	20	0.080	0.075	1.000	0.252
Hypercholesterolemia (% yes)	53	46	57	48	<0.001	<0.001	<0.001	1.000
CVD risk factors (0–4)	1.6 ± 1.0	1.5 ± 1.1	1.6 ± 1.0	1.5 ± 1.0	0.038	0.057	0.281	<0.001
MedDietScore (0–55)	29 ± 7.1	27 ± 6.8	27 ± 7.4	32 ± 5.4	<0.001	1.000	<0.001	<0.001
SAI (0–10)	3.0 ± 1.2	2.9 ± 1.3	3.0 ± 1.0	3.1 ± 1.4	0.064	0.581	0.335	0.093

Data are presented as mean values and SD or frequencies. *p*-values derived from analysis of variance (ANOVA) for continuous variables or the chi-square test for the categorical variables. *p* ^0^: comparisons between groups; *p* ^1^ : comparisons between “No culinary use of olive oil” and “Non-exclusive culinary use of olive oil”; *p* ^2^: comparisons between “Non-exclusive culinary use of olive oil” and “Exclusive culinary use of olive oil”; *p* ^3^: comparisons between “Exclusive culinary use of olive oil” and “No culinary use of olive oil”; after correcting for the inflation of Type-I error with the Bonferroni rule. BMI is body mass index, CVD is cardiovascular disease, SAI is successful aging index.

**Table 2:** Clinical and lifestyle characteristics of the participants, by age and type of culinary fat.

	50–70 Years Old				≥70 Years Old							
	Overall (*n* = 1707)	No Culinary Use of Olive Oil (*n* = 235)	Non-Exclusive Culinary Use of Olive Oil (*n* = 1017)	Exclusive Culinary Use of Olive Oil (*n* = 455)	Overall (*n* = 1642)	No Culinary Use of Olive Oil (*n* = 275)	Non-Exclusive Culinary Use of Olive Oil (*n* = 670)	Exclusive Culinary Use of Olive Oil (*n* = 697)	*p* ^0^	*p* ^1^	*p* ^2^	*p* ^3^
Male (%)	50	51	50	52	54	49 **^×^**	47 **^×^**	63 *^,†^	0.025	0.657	0.288	<0.001
Ever smokers (% yes)	49	44	52 *^,^**^×^**	44 ^†^	36	32 **^×^**	32 **^×^**	43 *^,†^	<0.001	0.006	<0.001	0.583
Physically active (% active)	41	32 **^×^**	38 **^×^**	52 *^,†^	42	35 **^×^**	33 **^×^**	53 *^,†^	0.698	0.405	0.015	0.659
BMI (kg/m^2^)	28 ± 4.6	29 ± 3.9	28 ± 4.7	28 ± 4.5	28 ± 4.3	28 ± 3.8	28 ± 4.4	28 ± 4.3	0.420	0.293	0.624	0.368
Hypertension (% yes)	80	85 **^×^**	84 **^×^**	69 *^,†^	91	92 **^×^**	96 **^×^**	86 *^,†^	<0.001	0.052	<0.001	<0.001
Diabetes (% yes)	17	21	15	17	25	28	27	23	<0.001	0.052	<0.001	0.015
Hypercholesterolemia (% yes)	54	42 ^†^	59 *^,^**^×^**	48 ^†^	51	51	54	48	0.084	0.048	0.025	0.915
CVD risk factors (0–4)	1.5 ± 1.1	1.3 ± 1.1 ^†^	1.5 ± 1.0 *	1.4 ± 1.1	1.7 ± 1.1	1.7 ± 1.1	1.8 ± 1.1 **^×^**	1.7 ± 1.0 ^†^	<0.001	<0.001	<0.001	<0.001
MedDietScore (0–55)	28 ± 7.1	26 ± 6.4 **^×^**	26 ± 6.8 **^×^**	32 ± 5.8 *^,†^	30 ± 6.8	28.4 ± 6.8 **^×^**	29 ± 7.9 **^×^**	32 ± 5.1 *^,†^	<0.001	<0.001	<0.001	0.867
SAI (0–10)	3.4 ± 1.1	3.3 ± 1.1	3.3 ± 0.9	3.4 ± 1.4	2.7 ± 1.3	2.3 ± 1.4 **^×^**	2.6 ± 1.0 **^×^**	3.0 ± 1.4 *^,†^	<0.001	<0.001	<0.001	<0.001

Values are presented as percent (%) or mean ± standard deviation. *p* ^0^: between 50–70 years old and over 70 years old comparisons, *p* ^1^: between 50–70 and over 70 for “No culinary use of olive oil” category, *p* ^2^: between 50–70 and over 70 for “Non-exclusive culinary use of olive oil” category, *p* ^3^: between 50–70 years old and over 70 years old for “Exclusive culinary use of olive oil” category, BMI = body mass index; SAI = successful aging index. *p*-values derived from Pearson’s chi-square test for categorical variables and from Pearson’s *t*-test for continuous variables. * *p*-value < 0.05 for the comparisons vs. “No culinary use of olive oil” category; ^†^ *p*-value < 0.05 for the comparisons vs. “Non-exclusive culinary use of olive oil” category and **^×^** *p*-value < 0.05 for the comparisons vs. “Exclusive culinary use of olive oil” category; after correcting for the inflation of Type-I error with the Bonferroni rule.

**Table 3:** Results from linear regression models (b ± SE) that evaluated the association between (a) “Non-exclusive culinary use of olive oil” category vs. “No culinary use of olive oil” category (independent variable) or (b) “Exclusive culinary use of olive oil” category vs. “No culinary use of olive oil” category (independent variable), and successful aging index (dependent outcome).

	Successful Aging Index	
	b ± SE	*p*
**Non-exclusive culinary use of olive oil vs. no culinary use of olive oil**		
Overall sample *	0.09 ± 0.06	0.114
50–70 years **	0.04 ± 0.07	0.570
≥70 years **	0.21 ± 0.12	0.093
**Exclusive culinary use of olive oil vs. no culinary use of olive oil**		
Overall sample *	0.33 ± 0.09	0.001
50–70 years **	0.08 ± 0.12	0.505
≥70 years **	0.38 ± 0.15	0.013

b: unstandardized B-coefficient, SE: Standard Error; * Adjusted for age, sex, smoking habits; ** Adjusted for sex and smoking habits.
